# Comparação das Novas Equações de Martin/Hopkins e Sampson para o Cálculo do Colesterol de Lipoproteína de Baixa Densidade em Pacientes Diabéticos

**DOI:** 10.36660/abc.20210641

**Published:** 2022-06-23

**Authors:** Abdulrahman Naser, Khagani Isgandarov, Tolga Sinan Güvenç, Rengin Çetin Güvenç, Müslüm Şahin

**Affiliations:** 1 Hospital Medical Park Pendik Departamento de Cardiologia Istanbul Turquia Hospital Medical Park Pendik, Departamento de Cardiologia, Istanbul – Turquia; 2 Universidade de Istanbul Faculdade de Medicina Cerrahpasa Departamento de Bioestatística e Informática da Medicina Istanbul Turquia Universidade de Istanbul – Faculdade de Medicina Cerrahpasa, Departamento de Bioestatística e Informática da Medicina, Fatih, Istanbul – Turquia; 3 Faculdade de Medicina da Istinye Divisão de Ciências Médicas Internas Departamento de Cardiologia Istanbul Turquia Faculdade de Medicina da Istinye, Divisão de Ciências Médicas Internas, Departamento de Cardiologia, Istanbul – Turquia; 4 Faculdade de Medicina da Okan Divisão de Ciências Médicas Internas Departamento de Cardiologia Istanbul Turquia Faculdade de Medicina da Okan, Divisão de Ciências Médicas Internas, Departamento de Cardiologia, Istanbul – Turquia

**Keywords:** Doenças Metabólicas, Aterosclerose, Dislipidemias, Doença da Artéria Coronariana, Diabetes Mellitus, Lipoproteína de Baixa Intensidade, LDL-Colesterol

## Abstract

**Fundamentos:**

A determinação precisa do colesterol de lipoproteína de baixa densidade (LDL-C) é importante para se alcançar concentrações de LDL-C recomendadas por diretrizes e para reduzir resultados cardiovasculares adversos em pacientes diabéticos. A equação de Friedewald comumente usada (LDL-Cf) produz resultados imprecisos em pacientes diabéticos devido a dislipidemia diabética associada. Recentemente, duas novas equações – Martin/Hopkins (LDL-CMH) e Sampson (LDL-Cs) – foram desenvolvidas para melhorar a precisão da estimativa de LDL-C, mas os dados são insuficientes para sugerir a superioridade de uma equação sobre a outra.

**Objetivos:**

O presente estudo comparou a precisão e a utilidade clínica das novas equações de Martin/Hopkins e Sampson em pacientes diabéticos.

**Método:**

Foram incluídos no estudo quatrocentos e dois (402) pacientes com diabetes. O risco cardiovascular dos pacientes e as metas de LDL-C foram calculadas por diretrizes europeias. As concentrações de LDL-Cmh, LDL-Cs, e LDL-Cf calculadas foram comparadas à concentração de LDL-C direto (LDL-Cd) para testar a concordância entre essas equações e LDL-Cd. Um P valor <0,05 foi aceito como estatisticamente significativo.

**Resultados:**

A LDL-CMH e a LDL-Cs tiveram concordância melhor com o LDL-Cd em comparação com a LDL-Cf, mas não houve diferenças estatísticas entre as novas equações para concordância com o LDL-Cd (Alfa de Cronbach de 0,955 para ambos, p=1). Da mesma forma, a LDL-CMH e a LDL-Cs tinham um grau semelhante de concordância com o LDL-Cd para determinar se o paciente estava dentro da meta de LDL-C (96,3% para LDL-Cmh e 96,0% para LDL-Cs), que eram ligeiramente melhores que a LDL-Cf (94,6%). Em pacientes com uma concentração de triglicérides >400 mg/dl, a concordância com o LDL-Cd foi ruim, independentemente do método usado.

**Conclusão:**

As equações de Martin/Hopkins e Sampson mostram uma precisão similar para o cálculo de concentrações de LDL-C nos pacientes com diabetes, e ambas as equações são ligeiramente melhores que a equação de Friedewald.

## Introdução

Existe uma relação bem conhecida entre colesterol de lipoproteína de baixa densidade (LDL-C) e doença arterial coronariana (DAC) aterosclerótica.^1^ Pacientes com diabetes não apenas têm maior probabilidade de ter DAC, mas também têm mais tendência a dislipidemias, incluindo triglicérides (TG) elevado, colesterol de lipoproteína de alta densidade (HDL-C), e maiores concentrações de partículas densas de LDL-C.^2 - 4^ Existem evidências fortes que sugerem resultados cardiovasculares melhores com tratamento de redução de colesterol em portadores de diabetes mellitus (DM) com dislipidemias, e, embora o relacionamento entre LDL-C e DCVs seja menos certo em pacientes com DM, as diretrizes internacionais disponíveis recomendam o uso de LDL-C como a meta principal para decisões de tratamento.^5 - 8^ Portanto, a medição precisa de LDL-C é primordial em pacientes com DM.

O padrão de ouro para medição de LDL-C é a β-quantificação, mas essa técnica é exigente tecnicamente e usa muitos recursos, por isso não é empregada rotineiramente na prática.^9^ Apesar de os ensaios de LDL-C direto (LDL-Cd) estarem comercialmente disponíveis agora, eles não são amplamente adotados, e muitos laboratórios ainda informam as concentrações de LDL-C calculado no lugar.^10^ A equação de Friedewald (LDL-Cf), que é o método mais comum empregado na prática, não é confiável quando a concentração de triglicérides ultrapassa 150 mg/dl e o LDL-C está abaixo de 70 mg/dl.^11 - 12^ Essa é uma preocupação particular para pacientes com DM, já que a hipertrigliceridemia é um componente comum da dislipidemia diabética. Recentemente, as equações de Martin/Hopkins (LDL-CMH) e de Sampson (LDL-Cs) foram desenvolvidas para dar uma melhor estimativa da concentração de LDL-C, especialmente quando o TG estiver elevado.^13 - 14^ Entretanto, poucos estudos apresentaram uma comparação direta dessas duas equações e não há dados em pacientes com DM.^15 - 17^

Na presente análise, o objetivo foi comparar as equações LDL-Cmh, LDL-Cs e LDL-Cf ao LDL-Cd para entender que equação tinha uma melhor concordância com LDL-Cd em pacientes diabéticos e até que ponto essas novas equações poderiam mudar a tomada de decisão clínica em comparação com a LDL-Cf.

## Materiais e métodos

### Seleção dos pacientes

Para a presente investigação, registros de pacientes cardiológicos ambulatoriais foram analisados retrospectivamente para os anos de 2019 e 2020. Pacientes com 18 anos de idade ou mais e portadores de diabetes no momento da internação foram incluídos no estudo. Pacientes cujos registros estavam incompletos foram excluídos. Nenhum outro critério de inclusão ou exclusão foi utilizado. Diabetes foi definido como a presença de um dos seguintes: i) estar em tratamento antidiabético com um diagnóstico anterior de diabetes ou ii) concentração de hemoglobina glicada igual ou maior que 6,5%. Dados demográficos, clínicos e laboratoriais dos pacientes foram coletados retrospectivamente a partir de um banco de dados eletrônico institucional. A taxa de filtração glomerular foi calculada usando-se a equação de dieta modificada em doença renal – taxa de filtração glomerular, e pacientes com uma taxa de filtração glomerular <60 ml/min/1,73 m^2^ foram aceitos como portadores de doença renal crônica. Os pacientes foram classificados como em risco cardiovascular intermediário, alto e muito alto, de acordo com as diretrizes europeias de 2019 sobre a gestão das dislipidemias.^7^ As metas de LDL-C para cada paciente individualmente foram determinadas usando as mesmas diretrizes. O estudo foi realizado de acordo com os princípios da Declaração de Helsinki de 1975 e suas revisões subsequentes, e a aprovação ética foi obtida de um comitê de ética local.

### Medição de LDL-C direto e o cálculo de LDL-C estimado

As amostras de sangue foram coletadas usando métodos padrão, e as amostras foram enviadas ao laboratório em até 30 minutos após a coleta. O LDL-Cd foi medido por um método colorimétrico usando o sistema de análise integrado Abbott Architect Plus ci8200 (Abbott Labs, Chicago, IL, EUA) e reagentes de teste Archem LDL-Cd (Archem Health Ind, Turquia).

Outras análises químicas de sangue, incluindo parâmetros lipídicos, foram realizados usando métodos-padrão e a mesma amostra de sangue foi usada para todas as análises. A LDL-Cf foi calculada como:


Eq1. LDL−C=CT−HDL−C−(TG/5)

conforme descrito anteriormente. Para o cálculo da LDL-Cs, foi utilizada a segunda equação relatada no trabalho de Sampson e colegas,^13^ que é a seguinte:


Eq2. $$\begin{array}{c} (CT/0,948)-(HDL-C/0,971)-(TG/8,56)+[(TG*\text{Não HDL-C / 2140) - (TG2 / 16100)]}-9,44 \end{array}$$

A LDL-Cmh precisa de um VLDL diferente: Os “fatores” de TG para cálculo em uma única equação matemática não podiam ser usados para derivar a LDL-CMH.^14^ Em vez disso, a LDL-CMH foi calculada usando planilhas disponibilizadas por um site mantido e apoiado pela Faculdade de Medicina da Johns Hopkins University.^18^

### Análises estatísticas

Variáveis contínuas foram expressas como média ± desvio padrão (DP) e variáveis categóricas foram apresentadas como porcentagem. Para as variáveis contínuas, os padrões de distribuição foram analisados pelo teste de Shapiro-Wilk e inspeção visual dos histogramas. Análises de correlação foram feitas com o teste de Pearson, e foram apresentados coeficientes de correlação para dar uma medida da força da relação entre métodos diferentes. Os gráficos de Bland-Altman foram elaborados para avaliar visualmente a concordância entre as concentrações de LDL-Cd e LDL-C calculado. Da mesma forma, o alfa de Cronbach e os coeficientes de correlação intraclasse foram calculados para a avaliação quantitativa da concordância. Os valores de alfa de Cronbach foram comparados usando-se o método de Feldt.^19^ A classificação correta para estar dentro da meta de LDL-C recomendada pelas diretrizes, bem como as taxas de reclassificação relativas a LDL-Cd, foram expressas em porcentagem. Os coeficientes de Kappa para a concordância foram calculados para cada par. Os pacientes foram estratificados por concentrações de TG (TG<150 mg/dl, TG 150-400 mg/dl, e TG>400 mg/dl) e análises de subgrupo separadas foram feitas para cada faixa. Por último, pacientes em uso de medicamentos anticolesterolêmicos foram analisados para entender a concordância entre as concentrações de LDL-Cd e LDL-C calculado em termos de alcançar a meta de concentração de LDL-C. Um p-valor <0,05 foi aceito como estatisticamente significativo para todas as comparações. As análises estatísticas foram realizadas com o Jamovi (The jamovi project (2020), Jamovi (Versão 1.2) para Windows. Retirados de (https://www.jamovi.org) e pacotes estatísticos SPSS 25.0 (IBM Corp, Armonk, NY, EUA).

## Resultados

As características demográficas e clínicas do grupo do estudo são apresentadas na Tabela 1 . Mais de quatro quintos da coorte do estudo tinham risco alto ou muito alto, enquanto apenas um quarto dos pacientes fazia uso de pelo menos um medicamento anticolesterolêmico. O LDL-C médio, calculado com todas as três equações, foi mais baixo que o LDL-Cd, e a maior diferença ocorreu entre o LDL-Cd e a LDL-Cf.

**Tabela 1:** Características demográficas, clínicas e laboratoriais da amostra do estudo

Característica	Valor		
Idade (anos)	56 ± 13		
Sexo (feminino)	189 (47,0%)		
Índice de massa corporal (kg/m^2^)	29,4 ± 4,4		
Pressão arterial sistólica (mmHg)	134,0 ± 17,5		
Pressão arterial diastólica (mmHg)	80,5 ± 10,1		
Tabagismo, n(%)	118 (29,4%)		
Doença arterial coronariana (%)	83 (20,6%)		
Doença renal crônica (%)	10 (2,7%)		
Antidiabéticos orais (%)	372 (92,5%)		
Insulina (%)	58 (14,4%)		
Medicamentos anti-hipercolesterolemia (%)	111 (27,6%)		
Glicemia jejum (mg/dl)	140,0 ± 54,1		
Hemoglobina glicada (%) (n=336)	7,0 ± 1,7		
Creatinina (mg/dl)	0,88 ± 0,24		
TFG (ml/min/m^2^)	90,4 ± 38,1		
Colesterol total (mg/dL)	199,0 ± 45,3		
Triglicérides (mg/dL)	163 (108 – 223)		
Colesterol HDL (mg/dl)	45,3 ± 10,6		
Colesterol LDL direto (mg/dl)	125,0 ± 35,0		
Faixa de risco SCORE			
Risco intermediário	75 (18,7%)		
Risco alto	212 (52,7%)		
Risco muito alto	115 (28,6%)		
Colesterol LDL Martin/Hopkins	120,0 ± 38,4		
Colesterol LDL Sampson	123,0 ± 38,1		
Colesterol LDL Friedewald	24,5 (7,6)		

*TFG: Taxa de filtração glomerular; HDL: Lipoproteína de alta densidade; LDL: lipoproteína de baixa densidade; ADO: Antidiabético oral; SCORE: Systematic coronary risk evaluation (Avaliação de risco coronário sistemática).*

### Correlação e concordância entre LDL-Cd e LDL-C calculado

**Figura 1:**
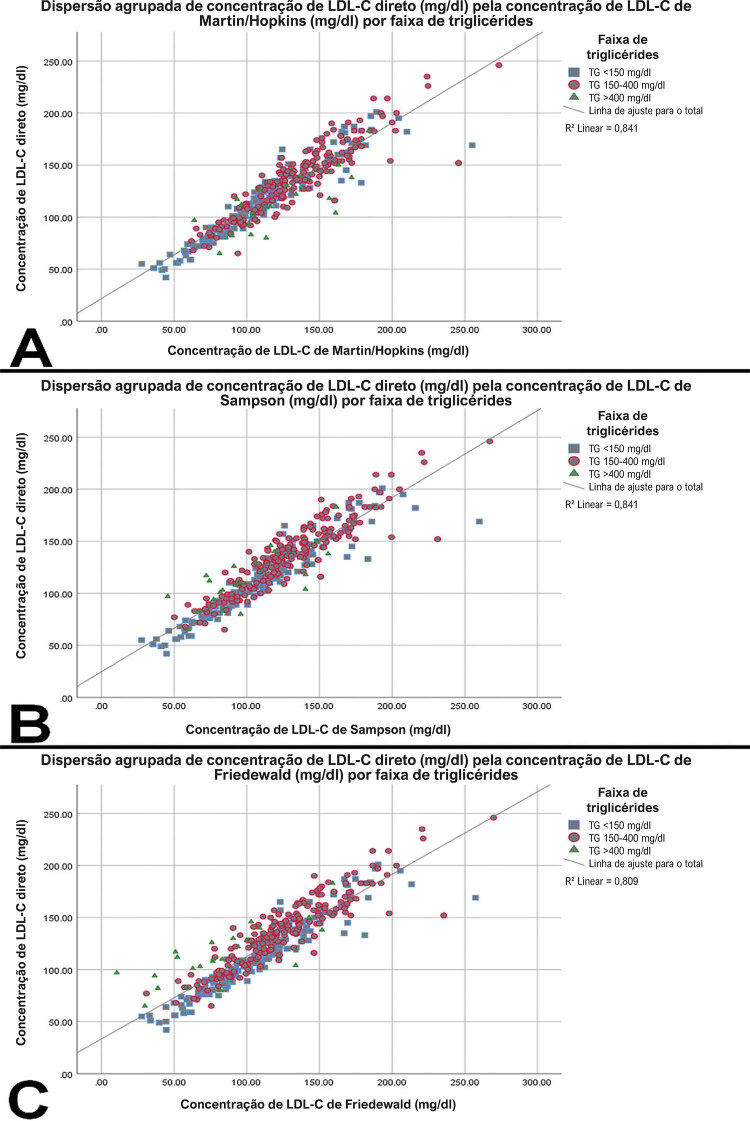
Gráficos de dispersão demonstrando a correlação de concentrações de colesterol LDL direto e concentrações de colesterol LDL calculado com (A) equação de Martin/Hopkins, (B) equação de Sampson e (C) equação de Friedewald. Os gráficos foram codificados por cores para refletir as concentrações de colesterol LDL em concentrações de triglicérides diferentes.

**Figura 2:**
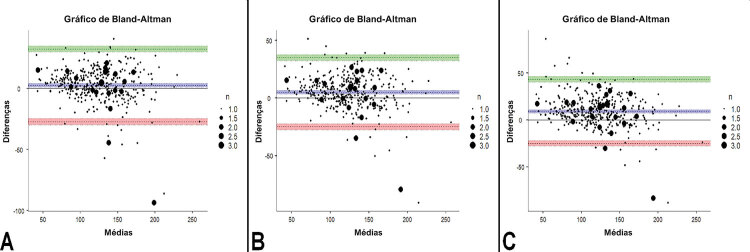
Gráficos de Bland-Altman demonstrando a concordância de concentrações de colesterol LDL direto e concentrações de colesterol LDL calculado com (A) equação de Martin/Hopkins, (B) equação de Sampson e (C) equação de Friedewald. As áreas coloridas nas partes superiores e inferiores dos gráficos mostram intervalos de confiança de 95% dos limites de concordância superiores e inferiores.

Todas as três equações apresentaram uma forte correlação com o LDL-Cd, mas a LDL-Cf tinha o valor mais baixo (r=0,915) em comparação com a LDL-Cmh (r=0,932) e a LDL-Cs (r=0,929) ( Figura 1 ). Dados sobre a concordância entre LDL-Cd e o LDL-C calculado foram apresentados na Tabela 2 . A LDL-Cmh e a LDL-Cs tinham uma concordância virtualmente similar com o LDL-Cd, enquanto ambas as equações tinham uma concordância significativamente melhor em comparação com a LDL-Cf (p<0,001 para ambos). Nos gráficos Bland-Altman, o número de casos que ultrapassaram os limites de concordância superior e inferior foi de 12 (2,98%) para LDL-Cmh, 15 (3,73%) para LDL-Cs, e 16 (3,98%) para LDL-Cf ( Figura 2 ).

**Tabela 2:** Concordância entre concentração de colesterol LDL direto e concentração de colesterol LDL calculado

Método	Alfa de Cronbach				ICC	
	alfa	p (vs. Martin)	p (vs. Sampson)	p (vs. Friedewald)	Coeficiente	IC 95%
Martin/Hopkins	0,955	-	1	<0,001	0,912	0,893 - 0,928
Sampson	0,955	1	-	<0,001	0,905	0,870 - 0,929
Friedewald	0,943	<0,001	<0,001	-	0,867	0,754 - 0,918

*IC: Intervalo de confiança; ICC: Coeficiente de correlação intraclasse.*

### Concordância e reclassificação

Dados sobre a concordância com o LDL-Cd por “estar dentro da meta de LDL-C”, bem como os índices de reclassificação, foram apresentados na Tabela 3 . A concordância foi semelhante para LDL-Cmh e LDL-Cs, e 3,7% e 3,9% dos casos podem ser reclassificados com o LDL-Cd, respectivamente. Os índices de reclassificação foram mais baixos com ambas as equações, quando comparados à LDL-Cf, já que o LDL-Cd reclassificou 5,5% dos casos que foram identificados dentro ou fora da meta de LDL-C pela LDL-Cf.

**Tabela 3:** Concordância entre o método de colesterol LDL direto e outros métodos para se alcançar as metas de colesterol LDL recomendadas por diretrizes

Método	Concordância	Subestimação	Superestimação	Kappa	p-valor
Martin/Hopkins	387 (96,3%)	12 (3,0%)	3 (0,7%)	0,774	<0,001
Sampson	386 (96,0%)	14 (3,4%)	2 (0,5%)	0,768	<0,001
Friedewald	380 (94,6%)	20 (5,0%)	2 (0,5%)	0,703	<0,001

*Concordância significa que ambos os métodos concordam se um paciente estava dentro ou fora da meta de colesterol LDL. Subestimação significa que o método em questão classificou casos como dentro da meta de colesterol LDL especificada, embora esses casos não atingissem a meta de colesterol LDL por métodos de colesterol LDL diretos. Superestimação significa que o método em questão classificou casos como fora da meta de colesterol LDL especificada, enquanto o método de colesterol LDL direto sugeria o contrário.*

### Concordância e reclassificação por faixa de TG

Em pacientes com TG <400 mg/dl; todas as três equações tiveram boa concordância com o LDL-Cd, mas a concordância foi ligeiramente melhor com a LDL-Cmh e a LDL-Cs se comparada à LDL-Cf ( Tabelas suplementares 1 e 2 ). A concordância foi razoavelmente melhor com a LDL-Cmh em pacientes dentro da faixa TG <150 mg/dl e com a LDL-Cs em pacientes com TG 150-400 mg/dl, mas as diferenças foram pequenas. Os índices de reclassificação também foram similares, embora a concordância com o LDL-Cd seja razoavelmente melhor com a LDL-Cmh do que com a LDL-Cs em pacientes com TG 150-400 mg/dl. É importante notar que o índice de reclassificação foi similar com a LDL-Cf quando comparado às novas equações nos pacientes com TG <150 mg/dl, mas não nos pacientes com TG >150 mg/dl.

A concordância entre LDL-Cd e equações novas foi ruim nos pacientes com concentração de TG acima de 400 mg/dl. A concordância por “estar dentro da meta” foi razoavelmente melhor para a LDL-Cs em comparação com a LDL-Cmh, embora a diferença fosse bastante trivial ( Tabela suplementar 2 ).

### Pacientes em tratamento anticolesterolêmico

Assim como em toda a coorte do estudo, os desempenhos da LDL-Cmh e da LDL-Cs foram semelhantes no subgrupo de pacientes em uso de medicamentos anticolesterolêmicos. É importante observar que ambas as equações tiveram uma concordância pequena, mas significativamente melhor, com o LDL-Cd, em comparação com a LDL-Cf, e os índices de reclassificação foram razoavelmente mais baixos quando a LDL-Cmh ou a LDL-Cs foram usadas em vez da LDL-Cf ( Tabelas suplementares 3 e 4 ).

### Pacientes com LDL-C <70 mg/dl

No presente estudo, 20 pacientes (4,9%) tinham um LDL-Cd <70 mg/dl, enquanto 33 (8,2%), 28 (7,0%) e 44 (10,9%) tinham LDL-C <70 mg/dl quando as equações de Sampson, Martin/Hopkins e Friedewald foram usadas. O número de pacientes classificados incorretamente como tendo LDL-C foi 15 (3,7%), 12 (3,0%) e 26 (6,4%) quando foram usadas LDL-Cs, LDL-Cmh e LDL-Cf, respectivamente. A Tabela suplementar 5 resume os índices de reclassificação com LDL-Cd para pacientes com um LDL-C calculado abaixo de 70 mg/dl. Os índices de reclassificação foram comparáveis para LDL-Cmh e LDL-Cs, mas proporcionalmente mais pacientes com LDL-Cf <70 mg/dl puderam ser reclassificados com LDL-Cd em comparação a pacientes com LDL-Cmh ou LDL-Cs <70 mg/dl.

## Discussão

No presente estudo, as concentrações de LDL-C calculadas foram comparadas ao LDL-Cd em pacientes diabéticos, com o foco específico na comparação de LDL-CMH e LDL-Cs, para se entender qual nova equação seria a mais útil clinicamente. Os principais aprendizados do presente estudo foram: i) LDL-CMH e LDL-Cs tinham uma relação forte e boa concordância com LDL-Cd, e não há grandes diferenças entre as equações em termos de reclassificação; ii) ambas as equações foram melhores que a LDL-Cf, especialmente em pacientes com TG>150 mg/dl, mas os benefícios de se usar qualquer uma das equações foram muito pequenos; iii) a LDL-Cmh teve uma concordância quase excelente com o LDL-Cd nos pacientes com uma concentração de TG entre 150-400 mg/dl, sendo que apenas 1,5% dos pacientes foram classificados erroneamente quando a LDL-Cmh era usada; iv) todas as equações tiveram um desempenho ruim quando as concentrações de TG ultrapassavam 400 mg/dl, sendo que menos de 90% dos pacientes puderam ser classificados corretamente, mesmo com a equação de melhor desempenho, a LDL-Cs; e v) a concordância entre LDL-Cd e LDL-C calculado foi ruim em pacientes com um LDL-C calculado abaixo de 70 mg/dl, sendo que mais de um quarto dos pacientes foi reclassificado como LDL-Cd, independentemente da equação usada. Ainda assim, nesse último subgrupo, a LDL-Cs e a LDL-Cmh tiverem um desempenho melhor que a LDL-Cf.

Com a possível exceção dos pacientes mais jovens com curta exposição a hiperglicemia, pacientes diabéticos têm alto risco de infarto do miocárdio e mortalidade coronária.^20^ Como a dislipidemia também é comum nesses pacientes, várias linhas de evidência sugerem que pacientes diabéticos se beneficiam de uma intensa redução de LDL-C com modificações no estilo de vida e o uso de medicamentos anti-hipercolesterolêmicos.^3 , 21 , 22^ Entretanto, o cálculo preciso do LDL-C é mais problemático em pacientes diabéticos considerando que níveis elevados de TG são comuns em diabéticos, e altas concentrações de TG levam à estimativa imprecisa do LDL-C. Isso é especialmente verdade para os cálculos feitos com a equação de Friedewald, que dá estimativas inadequadas de LDL-C quando as concentrações de TG estão acima de 150 mg/dl.^11^ As equações de Martin/Hopkins propiciam estimativas mais robustas de LDL-C e são menos sensíveis a mudanças em TG, desde que as concentrações de TG fiquem abaixo de 400 mg/dl.^14^ Mais recentemente, Sampson e colegas definiram uma nova equação, e seus achados iniciais sugerem que essa equação produz estimativas corretas de LDL-C desde que as concentrações de TG fiquem abaixo de 800 mg/dl.^13^ . Entretanto, ainda não havia certeza sobre até que ponto esses achados iniciais se aplicavam a pacientes diabéticos, ou se essas novas equações poderiam ter algum impacto no tratamento de pacientes. Um estudo recente que incluiu 1828 pacientes japoneses com diabetes detectou que as equações de Martin/Hopkins têm uma concordância melhor com o LDL-Cd e em comparação com a LDL-Cf, especialmente se os níveis de TG forem superiores a 150 mg/dl.^23^ Entretanto, esse estudo usou diretrizes japonesas para determinar se os pacientes estavam dentro das metas recomendadas por diretrizes, e, como as diretrizes japonesas não são amplamente usadas fora do Japão, a aplicabilidade de seus resultados para outras populações é incerta.^23^ Embora nossos resultados confirmem amplamente esse trabalho anterior, achados atuais também indicam que a concordância entre o LDL-C calculado e o LDL-Cd é superior a 90%, independentemente da equação usada, e, portanto, os benefícios clínicos do uso das novas equações de Martin/Hopkins ou Sampson, em vez da de Friedewald, são muito menos aparentes do que a hipótese inicial. Com isso em mente, considerando que ambas as equações permitem a classificação correta de uma proporção de casos significativamente mais alta praticamente sem custos adicionais (talvez com a exceção de incorporar equações mais complexas aos sistemas de automação existentes), o uso de qualquer uma das equações poderia ser recomendável em pacientes diabéticos.

Como as equações de Martin/Hopkins e de Sampson foram definidas nos últimos dez anos, estudos com comparações diretas dessas equações entre si ainda são raros. Dois estudos que compararam as equações de Martin/Hopkins e de Sampson à equação de Friedewald identificaram que sua capacidade de reclassificar casos era praticamente a mesma.^15 , 16^ Entretanto, esses estudos não compararam a precisão dessas equações em relação a um método de referência. Mais recentemente, Cwiklinska et al.,^17^ associados usaram tanto β-quantificação e um ensaio de LDL direto para comparar as equações de Martin/Hopkins e Sampson, e eles relataram que ambos os métodos eram mais precisos que a equação de Friedewald.^17^ Embora esse estudo não tenha oferecido uma comparação direta entre as duas novas equações, seus números indicam que o número de casos acima da meta de total de erros de 12% foi menor com a equação de Martin/Hopkins (134 vs. 157 casos).^17^ No entanto, esse estudo não relatou a possível importância clínica desses achados, e estes não foram específicos para pacientes com diabetes. Nossos resultados indicam que ambas as equações apresentaram uma concordância muito similar ao LDL-Cd e a tomada de decisão clínica deve ser semelhante na grande maioria dos pacientes, independentemente de que equação foi usada. Tendo dito isso, no subgrupo de pacientes com TG 150-400 mg/dl, a LDL-Cmh tinha uma concordância quase perfeita com o LDL-Cd em relação a estar “dentro da meta de LDL-C”, fazendo com que ela seja preferível para pacientes diabéticos nessa faixa de TG.

A estimativa do LDL-C se torna ainda mais difícil quando as concentrações de TG são superiores a 400 mg/dl, não apenas porque concentrações de lipoproteína de densidade muito baixa são subestimadas, mas também porque o LDL-C é suprimido aumentando-se os TG além desse ponto.^13^ Nem a equação de Friedewald, nem a de Martin/Hopkins apresentaram uma estimativa confiável de LDL-C acima do valor de corte.^10 , 24^ A equação de Sampson permitiu uma estimativa melhor do LDL-C para pacientes com hipertrigliceridemia com concentrações de TG de até 800 mg/dl, e, no estudo original, o índice de classificação indevida foi comparável ao da equação LDL-Cf para pacientes com TG <400 mg/dl.^13^ Uma nova equação promissora, que não foi incluída nesta análise, também foi apresentada recentemente para pacientes com doença renal crônica, nos quais a hipertrigliceridemia também é comum.^25^ Esta última equação parece ser tão precisa quanto a LDL-Cmh nesse subconjunto de pacientes, mas não foi validada para outros pacientes além dos portadores de doença renal.^26^ Na verdade, achados atuais não sugeriam a superioridade de uma equação nova em relação a outra. Nossos resultados indicaram que, apesar de a LDL-Cs ter a melhor concordância com o LDL-Cd, os índices de classificação indevida não eram aceitáveis, já que mais de 10% dos casos foram classificados indevidamente, independentemente da equação usada. Realmente, apenas um paciente adicional pode ser classificado corretamente quando a LDL-Cs foi usada em vez da LDL-Cmh ( Tabela suplementar 2 ). Portanto, o uso do LDL-Cd ou de um método alternativo como o colesterol não HDL-C ou concentrações de apolipoproteína b devem ser preferíveis em relação ao LDL-Cd estimado nesses pacientes, até que uma equação mais confiável esteja disponível.

Por último, sugere-se que a LDL-Cf tenha um desempenho ruim em pacientes com LDL-C <70 mg/dl devido a seus “fatores fixos”, e isso pode ser melhorado com as novas equações.^13 , 14^ Nossos achados indicam que a LDL-Cf classifica indevidamente até um terço dos pacientes diabéticos com um valor de LDL-Cf inferior a 70 mg/dl e esse número pode ser reduzido com o uso de novas equações, porém até um quarto desses pacientes ainda é classificado indevidamente como estando dentro das metas de tratamento mesmo com essas equações, sem diferenças importantes entre a LDL-Cs e a LDL-Cmh. Embora essa constatação corrobore o uso de novas equações em vez da LDL-Cf nesse subgrupo, ela, no entanto, sugere que nenhuma das equações disponíveis tem a confiabilidade adequada para pacientes diabéticos com LDL-C inferior a 70 mg/dl.

### Limitações do estudo

Ensaios enzimáticos diretos de LDL-C foram criticados por sua falta de confiabilidade e padronização, e a β-quantificação continua a ser o método padrão ouro para se quantificar o LDL-C^10^ . Entretanto, ensaios de última geração são muito mais confiáveis são endossados por diretrizes internacionais relevantes e testes enzimáticos de LDL-C já funcionaram como método de referência em vários estudos.^7 , 23 , 27 , 28^ A β-quantificação é muito trabalhosa para ser usada na prática de rotina, e mesmo a β-quantificação do LDL-C não está livre de erros, já que pode incluir colesterol de outras lipoproteínas.^13^ . A população do estudo era bem pequena (402 casos) e o número de casos com TG >400 mg/dl era de apenas 24, uma condição que pode ter afetado a confiabilidade da análise do subgrupo nessa faixa.

## Conclusões

Nos pacientes diabéticos, as equações de Martin/Hopkins e Sampson têm confiabilidade semelhante para a estimativa de LDL-C, sem vantagens óbvias de preferência de uma equação em relação à outra. Entretanto, ambas as equações foram superiores à equação de Friedewald em termos de concordância com o LDL-Cd e ambas tiveram índices de reclassificação mais baixos em comparação à LDL-Cf, especialmente em pacientes com TG>150 mg/dl. Como a diferença entre as duas equações foi trivial, qualquer uma das equações poderia ser preferida em relação à equação de Friedewald em pacientes diabéticos. No pequeno subgrupo de pacientes com concentração de TG acima de 400 mg/dl, nenhuma das equações teve precisão adequada, e, portanto, deve-se considerar a medição direta de LDL-C para esses pacientes.

## * Material suplementar

Para informação adicional, por favor, clique aqui


