# Supplementary material for: Comparação das Novas Equações de Martin/Hopkins e Sampson para o Cálculo do Colesterol de Lipoproteína de Baixa Densidade em Pacientes Diabéticos
Source: Arq Bras Cardiol. 2022 Jun 23;119(2):225–33. [Article in Portuguese] doi: 10.36660/abc.20210641 (PMC9363054; doi:10.36660/abc.20210641)
Supplement: Supplementary file 1 [file 2021-0641_AO_Supplementary_tables.pdf]

Supplementary Table 1. Agreement between direct LDL-cholesterol concentration and calculated LDL-cholesterol calculations per triglyceride stratum.

Method	Cronbach's alpha				ICC	
	alpha	p (vs. Martin)	p (vs. Sampson)	p (vs. Friedewald)	Coefficient	95% CI
Triglycerides <150 mg/dl (n=182)						
**Martin/Hopkins**	0.971	-	<0.001	<0.001	0.937	0.907 - 0.956
**Sampson**	0.968	<0.001	-	0.008	0.936	0.915 - 0.953
**Friedewald**	0.967	<0.001	0.008	-	0.930	0.897 - 0.951
Triglycerides 150-400 mg/dl (n=196)						
**Martin/Hopkins**	0.938	-	0.008	0.689	0.882	0.811 - 0.917
**Sampson**	0.943	<0.001	-	<0.001	0.877	0.911 - 0.917
**Friedewald**	0.937	0.689	<0.001	-	0.841	0.643 - 0.915
Triglycerides >400 mg/dl (n=24)						
**Martin/Hopkins**	0.858	-	0.408	0.021	0.735	0.479 - 0.876
**Sampson**	0.847	0.408	-	0.054	0.669	0.284 - 0.854
**Friedewald**	0.814	0.021	0.054	-	0.493	-0.70 - 0.790

CI: Confidence Interval; ICC: intraclass correlation coefficient.

Supplementary Table 2. Agreement between direct LDL-cholesterol method and other methods for reaching guideline-recommended LDL-cholesterol target per triglyceride stratum.

Method	Concordance	Underestimation	Overestimation	Kappa	P value
Triglycerides <150 mg/dl (n=182)					
**Martin/Hopkins**	174 (95.6%)	8 (4.4%)	0 (0.0%)	0.809	<0.001
**Sampson**	174 (95.6%)	7 (3.8%)	1 (0.5%)	0.801	<0.001
**Friedewald**	175 (96.1%)	6 (3.3%)	1 (0.5%)	0.823	<0.001
Triglycerides 150-400 mg/dl (n=196)					
**Martin/Hopkins**	193 (98.5%)	2 (1.0%)	1 (0.5%)	0.834	<0.001
**Sampson**	191 (97.5%)	4 (2.0%)	1 (0.5%)	0.749	<0.001
**Friedewald**	188 (95.9%)	7 (3.6%)	1 (0.5%)	0.646	<0.001
Triglycerides >400 mg/dl (n=24)					
**Martin/Hopkins**	20 (83.4%)	2 (8.3%)	2 (8.3%)	0.240	0.243
**Sampson**	21 (87.5%)	3 (12.5%)	0 (0.0%)	0.600	0.001
**Friedewald**	17 (70.8%)	7 (29.2%)	0 (0.0%)	0.333	0.028

Definitions are as before.

Supplementary Table 3. Agreement between direct LDL-cholesterol concentration and calculated LDL-cholesterol calculations in diabetic patients on antihypercholesterolemic treatment.

Method	Cronbach's alpha				ICC	
	alpha	p (vs. Martin)	p (vs. Sampson)	p (vs. Friedewald)	Coefficient	95% CI
**Martin/Hopkins**	0.972	-	0.44	0.0019	0.940	0.908 - 0.960
**Sampson**	0.973	0.44	-	<0.001	0.933	0.851 - 0.964
**Friedewald**	0.965	0.0019	<0.001	-	0.899	0.675 - 0.955

CI: Confidence Interval; ICC: intraclass correlation coefficient.

Supplementary Table 4. Agreement between direct low-density lipoprotein-cholesterol assay and other methods for reaching guideline-recommended target in diabetic patients on antihypercholesterolemic treatment.

Method	Concordance	Underestimation	Overestimation	Kappa	P value
**Martin/Hopkins**	106 (95.5%)	5 (4.5%)	0 (0.0%)	0.714	<0.001
**Sampson**	105 (94.6%)	6 (5.4%)	0 (0.0%)	0.673	<0.001
**Friedewald**	103 (92.8%)	8 (7.2%)	0 (0.0%)	0.703	<0.001

Definitions are as before.

Supplementary Table 5. Agreement between direct low-density lipoprotein-cholesterol assay and calculated low-density lipoprotein methods for reaching guideline-recommended target in diabetic patients with a calculated low density lipoprotein cholesterol less than 70 mg/dl.

Method	Concordance	Underestimation	Overestimation	Kappa	P value
**Martin/Hopkins LDL-C <70 mg/dl (n=28)**	20 (71.4%)	8 (28.6%)	0 (0.0%)	0.47	0.003
**Sampson LDL-C <70 mg/dl (n=33)**	24 (72.7%)	9 (27.3%)	0 (0.0%)	0.48	<0.001
**Friedewald LDL-C <70 mg/dl (n=44)**	28 (63.6%)	16 (36.4%)	0 (0.0%)	0.35	0.002

Definitions are as before. LDL-C, low-density lipoprotein cholesterol.
