# Supplementary material for: Comparação das Novas Equações de Martin/Hopkins e Sampson para o Cálculo do Colesterol de Lipoproteína de Baixa Densidade em Pacientes Diabéticos
Source: Arq Bras Cardiol. 2022 Jun 23;119(2):225–33. [Article in Portuguese] doi: 10.36660/abc.20210641 (PMC9363054; doi:10.36660/abc.20210641)
Supplement: Translation

# Translation: Comparison of Novel Martin/Hopkins and Sampson Equations for Calculation of Low-Density Lipoprotein Cholesterol in Diabetic Patients

**Authors:** Abdulrahman Naser, Khagani Isgandarov, Tolga Sinan Güvenç, Rengin Çetin Güvenç, Müslüm Şahin

**Affiliations:** 1 Turkey Medical Park Pendik Hospital, Department of Cardiology, Istanbul – Turkey; 2 Turkey Istanbul University-Cerrahpasa Faculty of Medicine, Department of Biostatistics and Informatics Medicine, Fatih, Istanbul – Turkey; 3 Turkey Istinye University Faculty of Medicine, Division of Internal Medical Sciences, Department of Cardiology, Istanbul – Turkey; 4 Turkey Okan University Faculty of Medicine, Division of Internal Medical Sciences, Department of Cardiology, Istanbul – Turkey

**Keywords:** Metabolic Diseases, Atherosclerosis, Dyslipidemias, Coronary Artery Disease, Diabetes Mellitus, Lipoproteins, LDL, Cholesterol, LDL

## Abstract

**Background:**

The accurate determination of low-density lipoprotein cholesterol (LDL-C) is important to reach guideline-recommended LDL-C concentrations and to reduce adverse cardiovascular outcomes in diabetic patients. The commonly used Friedewald equation (LDL-Cf), gives inaccurate results in diabetic patients due to accompanying diabetic dyslipidemia. Recently two new equations – Martin/Hopkins (LDL-Cmh) and Sampson (LDL-Cs) – were developed to improve the accuracy of LDL-C estimation, but data are insufficient to suggest the superiority of one equation over the other one.

**Objective:**

The present study compared the accuracy and clinical usefulness of novel Martin/Hopkins and Sampson equations in diabetic patients.

**Methods:**

This study included 402 patients with diabetes. Patients’ cardiovascular risk and LDL-C targets were calculated per European guidelines. Calculated LDL-Cmh, LDL-Cs, and LDL-Cf concentrations were compared with direct LDL-C concentration (LDL-Cd) to test agreement between these equations and LDL-Cd. A p-value <0.05 was accepted as statistically significant.

**Results:**

Both LDL-Cmh and LDL-Cs had a better agreement with LDL-Cd as compared to LDL-Cf, but no statistical differences were found among novel equations for agreement with LDL-Cd (Cronbach’s alpha 0.955 for both, p=1). Likewise, LDL-Cmh and LDL-Cs showed a similar degree of agreement with LDL-Cd in determining whether a patient was in a guideline-recommended LDL-C target (96.3% for LDL-Cmh and 96.0% for LDL-Cs), which were marginally better than LDL-Cf (94.6%). In patients with a triglyceride concentration >400 mg/dl, agreement with LDL-Cd was poor, regardless of the method used.

**Conclusion:**

Martin/Hopkins and Sampson’s equations show a similar accuracy for calculating LDL-C concentrations in patients with diabetes, and both equations were marginally better than the Friedewald equation.

## Introduction

There is a well-known relationship between low-density lipoprotein cholesterol (LDL-C) and atherosclerotic coronary artery disease (CAD).^1^Patients with diabetes are not only more likely to have CAD but are also more prone to dyslipidemias, including elevated triglycerides (TG), low high-density lipoprotein cholesterol (HDL-C), and increased concentrations of small, dense LDL-C particles.^2 - 4^There is strong evidence suggesting improved cardiovascular outcomes with cholesterol-lowering treatment in Diabetes Mellitus (DM) patients with dyslipidemias, and although the relationship between LDL-C and CAD is less certain in patients with DM, available international guidelines recommend using LDL-C as the primary target for management decisions.^5 - 8^Thus, accurate measurement of LDL-C is of paramount importance in patients with DM.

The gold standard for measuring LDL-C is β-quantification, but this technique is technically demanding and resource-intensive, so it is not routinely employed in practice.^9^While direct LDL-C (LDL-Cd) assays are now commercially available, these are not widely adopted, and many laboratories still report calculated LDL-C concentrations instead.^10^Friedewald equation (LDL-Cf), which is the most common method employed in practice, is unreliable when triglyceride concentration exceeds 150 mg/dl and LDL-C is below 70 mg/dl.^11 - 12^This is a particular concern for patients with DM, as hypertriglyceridemia is a common component of diabetic dyslipidemia. Recently, Martin/Hopkins (LDL-Cmh) and Sampson (LDL-Cs) equations were developed to provide a better estimate of LDL-C concentration, especially when TG is elevated.^13 - 14^However, few studies have provided a head-to-head comparison of these two equations, and there are no data in patients with DM.^15 - 17^

The present study aimed to compare LDL-Cmh, LDL-Cs, and LDL-Cf equations with LDL-Cd to understand which equation had a better agreement with LDL-Cd in diabetic patients and to what degree these novel equations could change clinical decision-making as compared to LDL-Cf.

## Materials and Methods

### Patient selection

For the present investigation, cardiology outpatient records were reviewed retrospectively for the years 2019 and 2020. Patients who were 18 years of age or older and had diabetes at the time of admission were included in the study. Patients with incomplete records were excluded. No other inclusion or exclusion criteria were used. Diabetes was defined as having one of the following: i) being on antidiabetic treatment with a previous diagnosis of diabetes or ii) a hemoglobin A1c% concentration equal to or greater than 6.5%. Patients’ demographic, clinical, and laboratory data were retrospectively collected from an institutional electronic database. Glomerular filtration rate was calculated using Modified Diet in Renal Disease – Glomerular Filtration Rate equation, and patients with a glomerular filtration rate <60 ml/min/1.73 m^2^were accepted as having chronic renal disease. Patients were classified into intermediate, high, and very-high cardiovascular risk according to the 2019 European guidelines on the management of dyslipidemias.^7^LDL-C targets for each individual patient were determined using the same guidelines. The study was conducted according to the principles of the 1975 Declaration of Helsinki and its subsequent revisions, and ethical approval was obtained from a local ethics committee.

### Measurement of direct LDL-C and calculation of estimated LDL-C

Blood samples were collected using standard methods, and samples were sent to the laboratory within 30 minutes after collection. LDL-Cd was measured by a colorimetric method using the Abbott Architect Plus ci8200 integrated analysis system (Abbott Labs, Chicago, IL, USA) and Archem LDL-Cd test reagents (Archem Health Ind, Turkey).

Other blood chemistry analyses, including lipid parameters, were carried out using standard methods, and the same blood sample was used for all analyses. LDL-Cf was calculated as:


Eq1. LDL−C=TC−HDL−C−(TG/5)

as previously described. To calculate LDL-Cs, the second equation reported in the work of Sampson et al. was used,^13^which is as follows:


Eq2. $$\begin{array}{c} (TC/0.948)-(HDL-C/0.971)-(TG/8.56)+[(TG*\text{Non-HDL-C / 2140) - (TG2 / 16100)]}-9.44 \end{array}$$

LDL-Cmh needs different VLDL: TG “factors” for calculation and a single mathematical equation could not be used to derive LDL-Cmh.^14^Instead, LDL-Cmh was calculated using spreadsheets provided by a supported and maintained website by Johns Hopkins University School of Medicine.^18^

### Statistical analyses

Continuous variables were given as mean ± standard deviation, while categorical variables were presented as percentages. For continuous variables, distribution patterns were analyzed with the Shapiro-Wilk test and visual inspection of the histograms. Correlation analyses were conducted by applying the Pearson test, and correlation coefficients were provided to give an overall measure of strength of relationship between different methods. Bland-Altman plots were drawn to visually assess the agreement between LDL-Cd and calculated LDL-C concentrations. Similarly, Cronbach’s alpha and intraclass correlation coefficients were calculated for a quantitative assessment of the agreement. Cronbach’s alpha values were compared using Feldt’s method.^19^Correct classification for being within the guideline-recommended LDL-C target, as well as reclassification rates relative to LDL-Cd was given as percentages. Kappa coefficients for the agreement were calculated for each pair. Patients were stratified per TG concentrations (TG<150 mg/dl, TG 150-400 mg/dl, and TG>400 mg/dl) and separate subgroup analyses were done for each stratum. Finally, patients on anticholesterolemic medications were analyzed to understand the agreement between LDL-Cd and calculated LDL-C concentrations in terms of reaching the target LDL-C concentration. A p-value <0.05 was accepted as statistically significant for all comparisons. Statistical analyses were performed with Jamovi (The jamovi project (2020). Jamovi (Version 1.2) for Windows, retrieved from (https://www.jamovi.org) and SPSS 25.0 (IBM Corp, Armonk, NY, USA) statistical packages.

## Results

The demographic and clinical characteristics of the study group were presented in Table 1 . More than four-fifths of the study cohort had either high or very high risk, while only a quarter of the patients were on at least one anticholesterolemic drug. Mean LDL-C calculated with all three equations were lower than LDL-Cd, while the largest difference was between LDL-Cd and LDL-Cf.

**Table 1:** Demographic. clinical. and laboratory characteristics of the study sample

Characteristic	Value		
Age (years)	56 ± 13		
Gender (female)	189 (47.0%)		
Body mass index (kg/m^2^)	29.4 ± 4.4		
Systolic blood pressure (mmHg)	134.0 ± 17.5		
Diastolic blood pressure (mmHg)	80.5 ± 10.1		
Smoking (%)	118 (29.4%)		
Coronary artery disease (%)	83 (20.6%)		
Chronic kidney disease (%)	10 (2.7%)		
Oral antidiabetic (%)	372 (92.5%)		
Insulin (%)	58 (14.4%)		
Antihypercholesterolemic drugs (%)	111 (27.6%)		
Fasting glucose (mg/dl)	140.0 ± 54.1		
Hemoglobin A1c (%) (n=336)	7.0 ± 1.7		
Creatinine (mg/dl)	0.88 ± 0.24		
GFR (ml/min/m^2^)	90.4 ± 38.1		
Total cholesterol (mg/dl)	199.0 ± 45.3		
Triglycerides (mg/dl)	163 (108 – 223)		
HDL-cholesterol (mg/dl)	45.3 ± 10.6		
Direct LDL-cholesterol (mg/dl)	125.0 ± 35.0		
SCORE risk strata			
Intermediate risk	75 (18.7%)		
High risk	212 (52.7%)		
Very high risk	115 (28.6%)		
Martin/Hopkins LDL-cholesterol	120.0 ± 38.4		
Sampson LDL-cholesterol	123.0 ± 38.1		
Friedewald LDL-cholesterol	24.5 (7.6)		

*GFR: Glomerular Filtration Rate; HDL: High-density lipoprotein; LDL: low-density lipoprotein; OAD: Oral antidiabetic; SCORE: Systematic coronary risk evaluation.*

### Correlation and agreement between LDL-Cd and calculated LDL-C

All three equations presented a strong correlation with LDL-Cd, but LDL-Cf showed the lowest value (r=0.915) compared to LDL-Cmh (r=0.932) and LDL-Cs (r=0.929) ( Figure 1 ). Data on the agreement between LDL-Cd and calculated LDL-C concentrations were presented in Table 2 . LDL-Cmh and LDL-Cs had a virtually similar agreement with LDL-Cd, while both equations had a significantly better agreement compared to LDL-Cf (p<0.001 for both). On Bland-Altman plots, the number of cases that exceeded upper and lower limits of agreement was 12 (2.98%) for LDL-Cmh, 15 (3.73%) for LDL-Cs, and 16 (3.98%) for LDL-Cf ( Figure 2 ).

**Figure 1:** Scatter plots showing the correlation of direct LDL-cholesterol concentrations with LDL-cholesterol concentrations calculated with (A) Martin/Hopkins equation, (B) Sampson equation, and (C) Friedewald equation. Plots were color-coded to reflect LDL-cholesterol concentrations at different triglyceride concentrations.

**Table 2:** Agreement between direct LDL-cholesterol concentration and calculated LDL-cholesterol concentrations

Method	Cronbach’s alpha				ICC	
	alpha	p (vs. Martin)	p (vs. Sampson)	p (vs. Friedewald)	Coefficient	95% CI
Martin/Hopkins	0.955	-	1	<0.001	0.912	0.893 - 0.928
Sampson	0.955	1	-	<0.001	0.905	0.870 - 0.929
Friedewald	0.943	<0.001	<0.001	-	0.867	0.754 - 0.918

*CI: Confidence Interval; ICC: intraclass correlation coefficient.*

**Figure 2:** Bland-Altman plots showing the agreement between direct LDL-cholesterol concentrations with LDL-choleserol concentrations calculated with (A) Martin/Hopkins equation, (B) Sampson equation, and (C) Friedewald equation. Colored regions at the upper and lower parts of the plots show 95% confidence intervals (CI) of upper and lower agreement limits.

### Concordance and reclassification

Data on agreement with LDL-Cd for “being in LDL-C target”, as well as reclassification rates, were provided in Table 3 . Concordances were similar for LDL-Cmh and LDL-Cs, and 3.7% - 3.9% of cases can be reclassified with LDL-Cd, respectively. Reclassification rates were lower with both equations as compared to LDL-Cf, as LDL-Cd reclassified 5.5% of the cases that were proved to be within or out of LDL-C target with LDL-Cf.

**Table 3:** Agreement between direct LDL-cholesterol method and other methods for reaching guideline-recommended LDL-cholesterol target

Method	Concordance	Underestimation	Overestimation	Kappa	p-value
Martin/Hopkins	387 (96.3%)	12 (3.0%)	3 (0.7%)	0.774	<0.001
Sampson	386 (96.0%)	14 (3.4%)	2 (0.5%)	0.768	<0.001
Friedewald	380 (94.6%)	20 (5.0%)	2 (0.5%)	0.703	<0.001

*Concordance means both methods agree whether a patient was within or out of the LDL-cholesterol target. Underestimation means that the method in question classified cases as within the specified LDL-cholesterol target, although these cases did not reach specific LDL-cholesterol target per direct LDL-cholesterol methods. Overestimation means that the method in question was classified as out of the specified LDL-cholesterol target while direct LDL-cholesterol method suggested otherwise.*

### Agreement and reclassification per TG strata

In patients with a TG <400 mg/dl, all three equations had a good agreement with LDL-Cd, but the agreement was slightly better with both LDL-Cmh and LDL-Cs as compared with LDL-Cf ( Supplementary Tables 1 and 2 ). The agreement was somewhat better with LDL-Cmh in patients within the TG <150 mg/dl strata and with LDL-Cs in patients with a TG 150-400 mg/dl, but the differences were minimal. Reclassification rates were also similar, although concordance with LDL-Cd was somewhat better with LDL-Cmh than LDL-Cs in patients with a TG 150-400 mg/dl. To note, the reclassification rate was similar with LDL-Cf when compared to novel equations in those with a TG <150 mg/dl, but not in those with a TG >150 mg/dl.

Concordance between LDL-Cd and novel equations was poor in those with a TG concentration above 400 mg/dl. Agreement for “being in target” was somewhat better for LDL-Cs as compared to LDL-Cmh, though the difference was rather trivial ( Supplementary Table 2 ).

### Patients on anticholesterolemic treatment

Similar to the whole study cohort, the performance of LDL-Cmh and LDL-Cs were similar in the subgroup of patients on anticholesterolemic drugs. To note, both equations had a small but significantly better agreement with LDL-Cd as compared to LDL-Cf, and reclassification rates were somewhat lower when either LDL-Cmh or LDL-Cs were used instead of LDL-Cf ( Supplementary Tables 3 and 4 ).

### Patients with an LDL-C <70 mg/dl

In the present study, 20 patients (4.9%) presented an LDL-Cd <70 mg/dl, while 33 (8.2%), 28 (7.0%), and 44 (10.9%) presented an LDL-C <70 mg/dl when Sampson, Martin/Hopkins, and Friedewald equations were used. The number of patients incorrectly classified as having an LDL-C were 15 (3.7%), 12 (3.0%), and 26 (6.4%) when LDL-Cs, LDL-Cmh, and LDL-Cf were used, respectively. Supplemental Table 5 summarizes reclassification rates with LDL-Cd for patients with a calculated LDL-C below 70 mg/dl. Reclassification rates were comparable for LDL-Cmh and LDL-Cs, but proportionally more patients with a LDL-Cf <70 mg/dl can be reclassified with LDL-Cd as compared to patients with LDL-Cmh or LDL-Cs <70 mg/dl.

## Discussion

The present study compared calculated LDL-C concentrations with LDL-Cd in diabetic patients, with a particular focus on comparing LDL-Cmh and LDL-Cs to understand which novel equation would be the most clinically useful. The main takeaways from the present study are: i) both LDL-Cmh and LDL-Cs had a strong relationship and a good agreement with LDL-Cd, and there are no major differences between equations in terms of reclassification; ii) both equations were better than LDL-Cf - especially in patients with a TG>150 mg/dl; however, the benefits of using either equation were marginal; iii) LDL-Cmh had a near-excellent concordance with LDL-Cd in those with a TG concentration between 150-400 mg/dl, with only 1.5% of the patients being misclassified when LDL-Cmh was used, iv) all equations performed poorly when TG concentration exceeded 400 mg/dl, with less than 90% of the patients being classified correctly even with the best-performing LDL-Cs equation; and v) agreement between LDL-Cd and calculated LDL-C was poor in those with a calculated LDL-C below 70 mg/dl, with more than a quarter of the patients being reclassified with LDL-Cd, regardless of the equation used. Nevertheless, in this latter subgroup, LDL-Cs and LDL-Cmh performed better than LDL-Cf.

With the possible exception of younger patients with a short exposure to hyperglycemia, diabetic patients are at high risk for myocardial infarction and coronary mortality.^20^As dyslipidemia is also common in these patients, multiple lines of evidence suggest that diabetic patients benefit from intensive LDL-C lowering with lifestyle modifications and antihypercholesterolemic drugs.^3 , 21 , 22^However, an accurate calculation of LDL-C is more problematic in diabetic patients, given that elevated TGs are common in diabetic patients and high TG concentrations cause an inaccurate estimation of LDL-C. This is especially true for calculations done with the Friedewald equation, which gives inadequate LDL-C estimates when TG concentrations are above 150 mg/dl.^11^Martin/Hopkins equations give more robust LDL-C estimates and are less sensitive to changes in TG, as long as TG concentrations are below 400 mg/dl.^14^More recently, Sampson et al. defined a new equation, and their initial findings suggest that this equation gives correct LDL-C estimates as long as TG concentrations are below 800 mg/dl.^13^However, to what extent were these initial findings applicable for diabetic patients, or whether these new equations could have any impact on patient management, were less certain. A recent study that included 1,828 Japanese patients with diabetes has found that Martin/Hopkins equations have a better agreement with LDL-Cd and as compared to LDL-Cf, especially when TG was above 150 mg/dl.^23^However, this study used Japanese guidelines to determine whether patients were within guideline-recommended targets, and as Japanese guidelines were not widely used outside Japan, the applicability of their results for other populations was uncertain.^23^While our results are largely confirmatory of this previous work, the present findings also indicate that the concordance between calculated LDL-C and LDL-Cd is above 90% regardless of the equation used; therefore, the clinical benefits of using novel Martin/Hopkins or Sampson equations over Friedewald equation is much less apparent than initially claimed. However, given that both equations allow correct classification of a significantly higher proportion of cases with virtually no additional costs (perhaps with the exception of incorporating more complex equations to the existing automation systems), using either equation could be advisable in diabetic patients.

Since both Martin/Hopkins and Sampson equations were defined in the last ten years, studies directly comparing these equations with each other are scarce. Two studies that compared Martin/Hopkins and Sampson equations with the Friedewald equation have found that their ability to reclassify cases was roughly similar.^15 , 16^However, these studies did not compare the accuracy of these equations against a benchmark method. More recently, Cwiklinska et al.^17^used both β-quantification and a direct LDL-assay to compare Martin/Hopkins and Sampson equations, and they reported that both methods were more accurate than the Friedewald equation.^17^While this study did not provide a head-to-head comparison between two novel equations, their numbers indicate that the number of cases exceeding the total error goal of 12% was smaller with Martin/Hopkins equations (134 vs. 157 cases).^17^Nonetheless, this study did not report the possible clinical importance of these findings, and their findings were not specific to patients with diabetes. Our results indicate that both equations had a very similar agreement with LDL-Cd and the clinical decision-making should be similar in the vast majority of patients regardless of which equation was used. Taking this into account, in the subgroup of patients with a TG 150-400 mg/dl, LDL-Cmh had a near-perfect agreement with LDL-Cd, thus making it preferable for diabetic patients in this TG strata.

Estimating LDL-C becomes even more difficult when TG concentrations exceed 400 mg/dl, not only because very-low-density lipoprotein concentrations are underestimated, but also because LDL-C is suppressed by increasing TGs beyond this point.^13^Neither the Friedewald nor the Martin/Hopkins equations gave a reliable estimate of LDL-C beyond that cut-off value.^10 , 24^The Sampson equation enabled a better estimation of LDL-C for patients with hypertriglyceridemia for TG concentrations up to 800 mg/dl, and in the original study, the misclassification rate was comparable to the misclassification rate of LDL-Cf equation for those with a TG <400 mg/dl.^13^A promising new equation, which was not included in this analysis, was also recently introduced for patients with chronic kidney disease, in whom hypertriglyceridemia is also common.^25^This latter equation appears to be as accurate as LDL-Cmh in this patient subset, but it was not validated beyond those with kidney disease.^26^Indeed, present findings were not suggestive of the superiority of one novel equation to another. Our results have indicated that while LDL-Cs had the best agreement with LDL-Cd, misclassification rates were unacceptable, as more than 10% of the cases were misclassified regardless of the equation used. Indeed, only one extra patient could be correctly classified when LDL-Cs were used instead of LDL-Cmh ( Supplementary Table 2 ). Therefore, using LDL-Cd or an alternative method, such as non-HDL-C cholesterol or apolipoprotein B concentrations, should be preferred over estimated LDL-Cd in these patients, until a more reliable equation is available.

Finally, it has been suggested that LDL-Cf performs poorly in patients with an LDL-C <70 mg/dl due to its “fixed factor”, and this can be improved with novel equations.^13 , 14^Our findings indicate that LDL-Cf misclassifies up to one-third of the diabetic patients with a LDL-Cf less than 70 mg/dl, and this figure can be lowered by applying novel equations, but up to one quarter of these patients are still misclassified as being within treatment targets even when using these equations, with no major difference between LDL-Cs and LDL-Cmh. Although this finding supports the use of novel equations rather than LDL-Cf in this subgroup, they nonetheless suggest that none of the available equations have adequate reliability for diabetic patients with an LDL-C below 70 mg/dl.

### Study Limitations

Direct enzymatic LDL-C assays have been criticized for a lack of reliability and standardization, and β-quantification remains as the gold standard method for quantifying LDL-C.^10^However, next-generation assays are much more reliable and are endorsed by relevant international guidelines, and enzymatic LDL-C assays have already served as the reference method in several studies.^7 , 23 , 27 , 28^β-quantification is too labor intensive to be used in routine practice, and even β-quantification of LDL-C is not devoid of errors, as it can include cholesterol from other lipoproteins.^13^The study population was rather small (402 cases), and the number of cases with a TG >400 mg/dl was only 24, a condition that might have affected the reliability of the subgroup analysis in this stratum.

## Conclusions

In diabetic patients, Martin/Hopkins and Sampson’s equations have similar reliability to estimate LDL-C, with no obvious advantage of preferring one equation over another. However, both equations were superior to the Friedewald equation in terms of agreement with LDL-Cd, and both had lower reclassification rates when compared to LDL-Cf, especially in patients with TG>150 mg/dl. Since the difference between the two equations was trivial, either equation could be preferred over the Friedewald equation in diabetic patients. In the small subset of patients with a TG concentration above 400 mg/dl, none of the equations had adequate accuracy and as such, direct measurement of LDL-C should be considered in these patients.

## * Supplemental Materials

For additional information, please click here
